# The *Biota orientalis,* oil extract Epiitalis^®^, is efficacious at reducing the symptoms of knee osteoarthritis: a pilot, multi-site, dose-ranging, randomized, blinded, placebo-controlled trial

**DOI:** 10.1007/s10787-022-01013-y

**Published:** 2022-06-22

**Authors:** Peter G. Mitchell, Corina A. Bright, Daniel R. Bright, Shalini N. Srivastava, Sonal S. Raote, Santosh Kumar

**Affiliations:** 1 Interpath Pty LTD, 10 Skipton Street, Ballarat, VIC 3350 Australia; 2 Vedic Lifesciences, Mumbai, India; 3 Independent Consultant Biostatistician, Delhi, India

**Keywords:** *Biota orientalis*, Knee osteoarthritis, Randomized placebo-controlled trial, VAS knee pain, WOMAC, SF-36

## Abstract

**Objective:**

To explore the safety, and efficacy of a proprietary hydrolyzed oil extract from seeds of *Biota orientalis* (hBO/Epiitalis^®^, Interpath Pty Ltd) in patients with knee pain due to osteoarthritis (OA).

**Methods:**

Patients aged 40–65 with X-ray diagnosed knee OA and knee pain ≥ 60 on a 100-point VAS (visual analog scale) were enrolled and randomized into four groups to receive daily hBO for 56 days as high (hBO-HD, 640 mg), mid (hBO-MD, 320 mg) or low (hBO-LD, 160 mg) doses, or a matched placebo oil. The primary outcome was change in VAS knee pain from baseline to 56 days in the mITT (modified intention to treat) population. Exploratory outcomes were the mWOMAC (modified Western Ontario and McMaster Universities Arthritis Index), and the SF-36 QoL (quality of life) questionnaire. The OMERACT-OARSI (Outcome Measures in Arthritis Clinical Trials–Osteoarthritis Research Society International) responder index was also calculated.

**Results:**

223 patients were included in the mITT population. Reductions in VAS scores between baseline and day 56 [Least square mean (LS mean) and 95% confidence interval (CI) of LS mean] were 36.4 (31.7–41.0), 37.9 (33.2–42.7), 35.7 (31.2–40.1) and 9.8 (14.5–15.2) for the hBO-HD, hBO-MD, hBO-LD, and placebo groups respectively. The VAS changes in all hBO groups were significantly different (*p* < 0.0001) vs. changes in the placebo group. hBO treatment led to similar quantitative beneficial changes in mWOMAC, SF-36 and OMERACT-OARSI responder index. There were no SAEs and no adverse events ascribed to the intervention.

**Conclusion:**

In a 56-day trial, hBO was safe, and was efficacious at reducing symptoms in patients with knee OA. Registration: NCT04117490; Oct 7, 2019.

## Introduction

Guidelines for the non-surgical management of osteoarthritis (OA) have recently been published by the Osteoarthritis Research Society International (OARSI) (Bannuru et al. 2019). With increasing understanding of the safety and/or efficacy issues of treatments such as opioids, acetaminophen, cyclooxygenase-2 (COX-2) inhibitors and non-selective anti-inflammatory drugs (NSAIDs), safe and effective options for oral chronic treatments for knee OA are becoming limited (Bannuru et al. 2019). In consequence, many OA patients with knee OA are left with limited therapeutic choices including non-pharmacological interventions, intra-articular (IA) injections such as hyaluronic acid (HA) or steroids, or ultimately joint replacement. Many patients have been exploring oral complementary and alternative interventions to treat their OA symptoms. These include chondroitin, glucosamine, collagen hydrolysates, curcumin, capsaicin, boswellia extracts, methylsulfonylmethane and others (Colletti and Cicero 2021; Liu et al. 2018). The most clinically researched of these are products containing chondroitin and/or glucosamine (Colletti and Cicero 2021); however, there is controversy in the literature regarding the efficacy of the products (Honvo et al. 2019; Reginster and Veronese 2021; Yang et al. 2021). Additional high-quality research is required to both confirm the activity of existing nutraceutical interventions for OA, and to identify new interventions that are safe and efficacious.

*Biota orientalis* (also called *Thuja orientalis* or oriental arborvitae) is a slow growing coniferous tree that is native to China and Iran. Extracts from the plant are commonly used in Chinese herbalism (Caruntu et al. 2020). Preclinical in vitro and *undefined* in vivo studies have demonstrated that extracts of *Biota orientalis* have antioxidant (Alamdari et al. 2017), and antiinflammatory (Jin et al. 2012; Kim et al. 2011, 2013; Lee et al. 2010; Shin et al. 2015) activities. Uniquely, the seed oils of gymnosperms contain relatively high concentrations of non-methylene-interrupted fatty acids (NMIFA). In the case of *Biota orientalis,* the most common NMIFA are (% total fatty acids): juniperonic (8.5–10.8%), sciadonic (3.6–4.4%), and keteleeronic (0.7–0.9%) (Wolff et al. 1996). In vitro, both sciadonic acid (Chen et al. 2015, 2012) and juniperonic acid (Tsai et al. 2018) can reduce the expression of inflammatory mediators in murine RAW264.7 macrophages (Chen et al. 2012; Tsai et al. 2018) and/or murine Microglial BV-2 Cells (Chen et al. 2015). In vitro, treatment with sciadonic acid, reduced prostaglandin E2 (PGE2), the expression of cyclooxygenase-2 (COX-2) mRNA, and the relative expression of activated mitogen activated protein kinases (MAPK) (Chen et al. 2015, 2012). Additionally, it was demonstrated that NMIFA are incorporated into membrane lipids and replace proinflammatory fatty acids such as arachidonic acid (Chen et al. 2015, 2012; Tsai et al. 2018). Mechanistically, it is hypothesized that the substitution of membrane lipids with NMIFA reduces substrate availability for COX-2 and modulates MAPK signaling, resulting in an overall reduction in the response to inflammatory stimuli (Chen et al. 2015, 2012).

In vitro, simulated gastric digests containing hBO reduced interleukin-1 (IL-1) stimulated PGE2 production and increased chondrocyte viability in cartilage explants (Pearson et al. 2008), while an oral product containing hBO reduced synovial fluid PGE2 and glycosaminoglycan (GAG) levels resulting after intraarticular IL-1β injections in horses (Pearson et al. 2009). Additionally, the oral product containing hBO also significantly reduced synovial fluid PGE2 in horses after surgical removal of osteochondral fragments (Pearson et al. 2012). The efficacy of a commercial product containing hBO (4CYTE™ Canine, Interpath Pty Ltd) was recently assessed in a randomized, blinded trial in dogs with naturally occurring OA in which hBO was demonstrated to be non-inferior to carprofen vs. pain and function endpoints (Whittem et al. 2021). Additionally, in dogs with naturally occurring OA, hBO as a stand-alone intervention was effective at reducing both objective and subjective endpoints relating to joint pain and function (Beths et al. 2020). In both canine clinical OA studies, the efficacy of hBO had a relatively slow onset, reaching a maximum at study termination (one month). Finally, in a pilot 70-day equine model of surgically induced OA, hBO significantly reduced synovial fluid PGE2 concentration and white blood cell number, as well as significantly reduced the progression of X-ray measured joint scores (Seabaugh et al. 2022).

Based on these promising in vitro, in vivo, and canine clinical studies, a pilot, multi-site, dose-ranging, randomized, blinded, placebo-controlled human trial of hBO was initiated. The objective of this study was to explore the safety, and efficacy of hBO in OA patients with knee pain. The primary outcome was set a priori as change in the pain visual analog score (VAS) from baseline to day 56 in comparison to the change in pain VAS in the placebo group.

## Methods

### Study design

The study was a pilot, multi-site, dose-ranging, randomized, blinded, placebo-controlled trial sponsored by Interpath and conducted by Vedic Lifesciences (Mumbai, India). All study participants provided written informed consent. Six clinical study sites in Mumbai, India were utilized in the trial conducted between December 2019 and August 2020. Training of investigators and study staff during site initiation, and on the day of first participant randomization was conducted by Vedic personnel. All study-related data were captured via electronic Clinical Report Forms (eCRF). A data management team conducted verification of the source data and e-CRF, with follow-up Quality Assurance checks. At study entry, patients were randomly allocated into four groups: 3 different doses of hBO, plus a placebo group. Screening was conducted between days −12 to −8, randomization was at the baseline visit (day 0), with follow-up visits on days 14 (± 1; Visit I), 28 (± 2, Visit II) and at study end (day 56 ± 2; Visit III). Study medication was dispensed at baseline and at Visit II, with compliance assessed at Visits I and II, and at study end. Anti-inflammatory, analgesic therapies and nutraceuticals for arthritis were prohibited during the study with the exclusion of acetaminophen, which served as a rescue medication. Serum samples for the assessment of safety-related biochemical markers were taken from each participant at the screening visit, and at study end.

All study data was to be collected in-person at each study visit; however, due to the COVID-19 outbreak midway through the study, participants were instead dispensed with study related questionnaires and responses collected digitally through video conferencing. Blood sample collection, dispensing of study medications and study dairies were carried out at each participant’s home post the COVID-19 outbreak. An interim analysis was conducted to assess for any differences in the baseline data (the only time point available with sufficient data) when comparing pre and post-COVID-19 outbreak randomization. No significant differences were detected, providing support that the COVID-19 outbreak and resulting changes in study procedures were unlikely to affect study outcomes.

### Study population

Major inclusion criteria included: (1) male & female aged ≥ 40 to ≤ 65 years suffering from primary idiopathic osteoarthritis of the knee diagnosed at least 3 months prior to screening; (2) Kellgren Lawrence (KL) scores of II or III; (3) pain in their index (worst) knee rated ≥ 60 on a 100-point VAS. Major exclusion criteria included: (1) a history of OA for > 3 years; (2) a history of osteoporosis and/or frequent fractures; (3) major trauma or arthroscopic surgery to the index joint; (4) patients who had received IA steroids or HA injections within the last three months; (5) patients waiting for joint replacement surgery; (6) Unwillingness to abstain from the use of NSAIDs, immunosuppressives, or joint nutraceutical supplements; (7) history of any immune or inflammatory disease, or any major chronic hepatic, cardiovascular, neurological, malignant or immunosuppressive condition, or the presence of any infection. A 7-day placebo run-in was initiated for each prospective participant at the screening visit. Prospective participants were dispensed bottles of placebo and instructed to take morning and night. At the randomization visit, patients were excluded if (1) they had ≥ 10 mm reduction in their VAS pain score compared to their screening visit), or (2) had modified Western Ontario and McMaster Universities Arthritis Index (mWOMAC) pain scores < 13, or (3) had < 80% compliance during the placebo run-in period.

### Randomization and blinding

Stratified block randomization using blocks of 8 was performed using Stats direct software version 3.1.17. At day 0, participants were randomized in the ratio 1:1:1:1 into either of the four treatment arms. Active and placebo capsules were matched for size, shape, colour and texture then packaged in identical plastic bottles. Blinding was performed at a Vedic-approved facility with blinding codes secured in tamper-evident sealed envelopes, each stating the participant identification (ID) and the study product allocation (active or placebo), all maintained in the trial master file. A secured soft copy back-up of the master randomization chart, accessible to designated personnel only, was stored in a folder under the Contract Research Organization (CRO) local area network. The patients, sponsor, CRO lead investigators, staff responsible for investigational product (IP) dispensing, site investigators and statistician were all blinded to study treatment until the database was locked.

### Intervention

The four study groups consisted of: high dose (hBO-HD; 320 mg twice daily [bid]), mid dose (hBO-MD, 320 mg once daily [qd]), low dose (hBO-LD, 160 mg qd), and placebo. Seeds from *Biota orientalis* are sourced in China and harvested under contract to Interpath Pty Ltd. Hydrolysis of cold pressed seed oil is carried out via a proprietary method by Callaghan Innovation (Lower Hutt, New Zealand). To ensure consistency, each batch of hBO undergoes an analytical QA that includes assessment of the composition and quantity of all major fatty acids plus assessment of the oxidation status. All batches of hBO must meet predetermined analytical QA criteria prior to acceptance. Capsules of hBO and placebo were prepared by Arbro Pharmaceuticals Ltd (New Delhi, India). Placebo capsules contained coconut-derived medium chain triglyceride oil (MCT; Dubois-Natural Esters, Malaysia) that was matched for composition and colour with hBO. Each participant received four bottles containing their treatment regimen with dosing instructions as per Table 1. Records were maintained of all IP administered to each participant, and by each participant in their accountability log. Compliance was required to be a minimum of 90% during the entire study period and was checked at each visit.

**Table 1:** Treatment regimens

Group	Daily dose	Morning regimen	Evening regimen
hBO-LD	160 mg qd	One 160 mg hBO capsule + one 160 mg placebo capsule	Two 160 mg placebo capsules
hBO-MD	320 mg qd	Two 160 mg hBO capsules	Two 160 mg placebo capsules
hBO-HD	320 mg bid	Two 160 mg hBO capsules	Two 160 mg hBO capsules
Placebo (MCT)	320 mg bid	Two 160 mg placebo capsules	Two 160 mg placebo capsules

### Efficacy outcomes

The prespecified primary efficacy outcome was change in pain in the index knee from baseline to day 56 using a 100-point VAS scale (da Costa et al. 2021). Exploratory efficacy outcomes were: (1) Changes from day 0 to day 56 in knee pain, function and stiffness utilizing a modification to the WOMAC scale (mWOMAC) validated for use in Indian and Asian populations (Bellamy et al. 1988). (2) Changes from day 0 to day 56 in the Medical Outcome Study Short Form-36 (SF-36) Quality of Life (QoL) questionnaire (Patel et al. 2007).

Since VAS pain data were also collected on study days 14 and 28, VAS pain scores were additionally plotted at each study time point to visualize the kinetics of efficacy onset. Finally, assessment of the percentage of responders and non-responders at each study time point was calculated as per the OMERACT–OARSI responder index (Pham et al. 2004).

### Safety

Blood pressure and heart rate were taken at each patient visit. Serum biochemical markers of liver (AST, aspartate aminotransferase; ALT, alanine aminotransferase; ALP, alkaline phosphatase) and kidney (creatinine) function were assessed from blood samples taken at the screening visit and at study end. The occurrence of adverse events (AEs) and serious adverse events (SAEs) were monitored throughout the study period.

### Sample size

Sample size for this pilot trial of hBO was based on assumptions and power calculations from a previous trial assessing knee pain with VAS as the primary outcome (Hughes and Carr 2002). In summary, this power calculation indicated that 38 patients/group would be required to detect a 20 mm difference in VAS pain score with a 90% power at 1% significance.

A total of 235 research participants were randomized to obtain approximately 200 completed participants after estimating a 15% dropout rate and a target of ≈ 50/group.

### Statistical methods

Changes in scores (baseline to each time point) for the VAS, WOMAC and SF-36 data were visually assessed for normality and in each case determined to reasonably approximate normal distributions. Analysis of Variance (ANOVA) models utilized the change-from-baseline of each outcome (primary and exploratory) as dependent variables with treatments as fixed effects. The estimates were presented as LS Means and associated 95% confidence intervals (CI) based on an ANOVA model. For the primary outcome, pairwise comparisons were performed using t-tests with a Bonferroni correction to correct for testing multiplicity. For the exploratory outcomes, pairwise comparisons were performed using t-tests; however, no adjustments for testing multiplicity were made and these *p* values are regarded as nominal. On day 56, a chi-square test was utilized to assess for differences between the distribution of OMERACT-OARSI responders in the hBO groups vs. the placebo group.

## Results

### Characteristics of the randomized trial participants

**Fig. 1:**
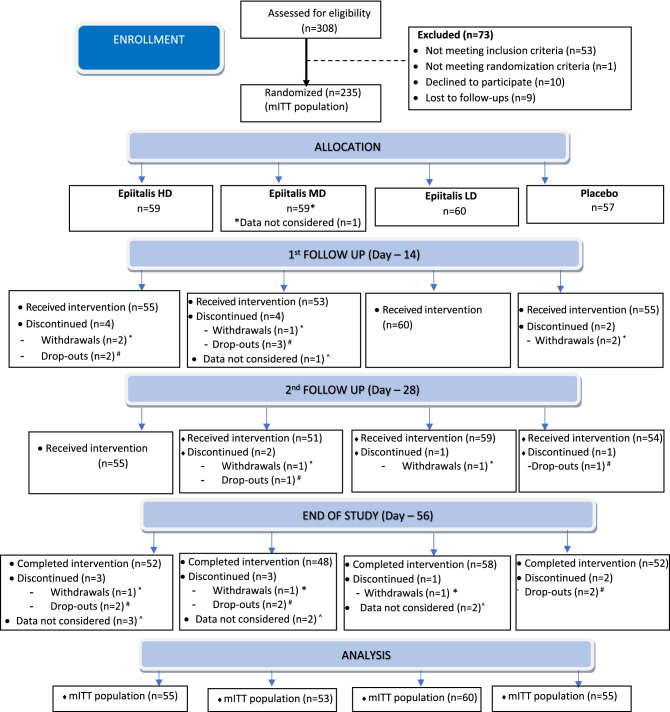
Trial profile. *Withdrawal = participants who withdrew consent during the study. #Dropout = participants who were not contactable or who did not turn up for a follow-up visit. ^Data not considered = any protocol violation or major protocol deviation finding during the study period

A total of 308 potential participants were screened with 235 randomized (Fig. 1). 234 participants were included in the safety population, out of which 223 were included in the modified intention to treat (mITT) population, defined as all participants who took at least one dose of the IP and at least completed the day 14 visit. Key baseline demographic characteristics, as well as VAS pain and the proportion of KL II and III scores are outlined in Table 2. The mean (min, max) age of participants was 51.7 (40, 65) years, 63.3% were women and their mean (min, max) BMI was 26.1 kg/m^2^ (20.7, 29.8). Age, gender, BMI, distribution of KL II/II grades, and baseline knee VAS were similar across all treatment groups at baseline. Compliance was assessed to be > 99% for each of the four IP bottles for each study group, and at each study time point.

**Table 2:** Baseline demographic characteristics of study participants

	hBO-HD (*n* = 59)	hBO-MD (*n* = 58)	hBO-LD (*n* = 60)	Placebo (*n* = 57)	Total (*n* = 234)
Gender n (%)					
Male	19 (32.20%)	19 (32.76%)	21 (35.00%)	27 (47.37%)	86 (36.75%)
Female	40 (67.80%)	39 (67.24%)	39 (65.00%)	30 (52.63%)	148 (63.25%)
Age (years)					
Mean	52	52.02	50.33	52.35	51.66
Median	51	52.5	49	54	51.5
(Min, max)	(40.00, 64.00)	(40.00, 64.00)	(40.00, 65.00)	(40.00, 65.00)	(40.00, 65.00)
BMI (Kg/m^2^)					
Mean	26.33	26.03	25.98	25.96	26.08
Median	26.53	25.87	26.34	26.06	26.23
(Min, max)	(21.79, 29.78)	(22.15, 29.69)	(20.69, 29.74)	(21.22, 29.68)	(20.69, 29.78)
Kellgren And Lawrence					
Grade-II n (%)	49 (83.05%)	49 (84.48%)	50 (83.33%)	49 (85.96%)	197 (84.19%)
Grade-III n (%)	10 (16.95%)	9 (15.52%)	10 (16.67%)	8 (14.04%)	37 (15.81%)
Pain VAS					
Mean	78.31	78.1	75.5	78.25	77.52
Median	80	80	70	80	80
(Min, max)	(60.00, 100.00)	(60.00, 100.00)	(60.00, 100.00)	(60.00, 100.00)	(60.00, 100.00)

### Primary endpoint

Table 3 outlines the primary outcome data i.e., changes in pain VAS (Baseline to day 56) in the hBO groups vs. the corresponding change in the placebo group on day 56. The change in absolute VAS scores (day 0–day 56) were (LS mean [95% C.I.]) − 36.4 (− 41.0, − 31.7), − 37.9 (− 42.7, − 33.2), − 35.7 (− 40.1, − 31.2) and − 9.8 (− 14.5, − 5.2) in the hBO-HD, hBO-MD, hBO-LD and placebo groups, respectively. Between group analyses demonstrated that the pain VAS changes in each hBO group were completely separated (LS mean and 95% CI) and statistically different (all *p* < 0.0001) vs. the change in pain VAS in the placebo group. Although the data did not demonstrate a dose response relationship, at study end each hBO dose resulted in robust and highly significant reductions in knee pain in comparison to the placebo group.

**Table 3:** Change in Pain VAS from day 0 to day 56 (primary endpoint) in the mITT population

	Group			
VAS Pain	HD-hBO (*n* = 55)	MD-hBO (*n* = 53)	LD-hBO (*n* = 60)	Placebo (*n* = 55)
Day 0–day 56				
LS mean	− 36.4	− 37.9	− 35.7	− 9.8
LS mean 95% CI	(− 41.0, − 31.7)	(− 42.7, − 33.2)	(− 40.1, − 31.2)	(− 14.5, − 5.2)
*p* value	< 0.0001	< 0.0001	< 0.0001	

Least square [LS] means and 95% confidence interval [CI] of the LS means. Pair wise comparisons utilized *t*-tests with Bonferroni correction. *p *Values for each hBO group vs. the placebo group

### Exploratory endpoints

For the following exploratory endpoints, no corrections were made for multiple comparisons with conclusions regarded as hypothesis generating.

For each mWOMAC subdomain, and for the mWOMC total, between group comparisons demonstrated that the changes in each hBO group were completely separated (LS mean and 95% CI), and statistically different (all *p* < 0.0001) vs. the change in the placebo group (Table 4).

**Table 4:** Change in exploratory endpoints day 0–day 56: mWOMAC and SF-36 (Least square [LS] means and 95% confidence interval [CI] of the LS means)

Endpoint	Group			
	HD-hBO (*N* = 55)	MD-hBO (*N* = 53)	LD-hBO (*N* = 60)	Placebo (*N* = 55)
mWOMAC pain change				
LS mean	− 7.9	− 8.2	− 8	− 1.8
LS mean 95% CI	(− 9.0, − 6.8)	(− 9.3, − 7.1)	(− 9.0, − 7.0)	(− 2.9, − 0.7)
Nominal *p* value	< 0.0001	< 0.0001	< 0.0001	
mWOMAC stiffness change				
LS mean	− 2.6	− 2.6	− 2.4	− 0.8
LS mean 95% CI	(− 3.1, − 2.0)	(− 3.2, − 2.1)	(− 2.9, − 1.9)	(− 1.3, − 0.2)
Nominal *p* value	< 0.0001	< 0.0001	< 0.0001	
mWOMAC function change				
LS mean	− 26	− 27.8	− 24.7	− 5
LS mean 95% CI	(− 30.1, − 21.9)	(− 31.9, − 23.6)	(− 28.6, − 20.8)	(− 9.0, − 0.9)
Nominal *p* value	< 0.0001	< 0.0001	< 0.0001	
mWOMAC total change				
LS mean	− 36.5	− 38.6	− 34.3	− 7.5
LS mean 95% CI	(− 42.0, − 30.9)	(− 44.3, − 33.0)	(− 39.6, − 29.0)	(− 13.0, − 2.0)
Nominal *p* value	< 0.0001	< 0.0001	< 0.0001	
SF-36 Physical function change				
LS mean	35.8	38.2	36.2	3.1
LS mean 95% CI	(29.1, 42.5)	(31.3, 45.0)	(29.8, 42.6)	(− 3.6, 9.8)
Nominal *p* value	< 0.0001	< 0.0001	< 0.0001	
SF-36 physical health change				
LS mean	62.7	56.6	60.8	2.7
LS mean 95% CI	(51.5, 74.0)	(45.2, 68.1)	(50.1, 71.6)	(− 8.5, 14.0)
Nominal *p* value	< 0.0001	< 0.0001	< 0.0001	
SF-36 body pain change				
LS mean	32	35.3	32.5	2.9
LS mean 95% CI	(25.2, 38.8)	(28.4, 42.3)	(26.0, 39.1)	(− 3.9, 9.7)
Nominal *p* value	< 0.0001	< 0.0001	< 0.0001	
SF-36 social function change				
LS mean	27.3	31.1	25.2	1.6
LS mean 95% CI	(20.9, 33.7)	(24.6, 37.6)	(19.1, 31.3)	(− 4.8, 8.1)
Nominal *p* value	< 0.0001	< 0.0001	< 0.0001	
SF-36 mental function change				
LS mean	18.3	21	18.1	− 1.8
LS mean 95% CI	(12.7, 23.9)	(15.3, 26.7)	(12.8, 23.5)	(− 7.4, 3.9)
Nominal *p* value	< 0.0001	< 0.0001	< 0.0001	
SF-36 emotional health change				
LS mean	45.5	43.4	42.8	− 0.6
LS mean 95% CI	(32.6, 58.3)	(30.3, 56.5)	(30.5, 55.1)	(− 13.5, 12.3)
Nominal *p* value	< 0.0001	< 0.0001	< 0.0001	
SF-36 vitality change				
LS mean	20.2	21.7	20.9	− 0.5
LS mean 95% CI	(14.2, 26.2)	(15.6, 27.8)	(15.2, 26.6)	(− 6.4, 5.5)
Nominal *p* value	< 0.0001	< 0.0001	< 0.0001	
SF-36 general health change				
LS mean	16.4	18.4	17.8	2.5
LS mean 95% CI	(11.5, 21.3)	(13.4, 23.4)	(13.2, 22.5)	(− 2.4, 7.4)
Nominal *p* value	0.0003	< 0.0001	< 0.0001	
SF-36 total score change				
LS mean	258.1	265.7	254.4	10.9
LS mean 95% CI	(210.5, 305.6)	(217.2, 314.1)	(208.9, 299.9)	(− 36.6, 58.5)
Nominal *p* value	< 0.0001	< 0.0001	< 0.0001	

Pair wise comparisons (hBO group vs. placebo) utilized *t* tests without multiplicity correction and *p *values are presented as nominal

Similarly for the total SF-36 score and for each component of the SF-36, which includes physical and mental well-being domains, between group comparisons demonstrated that the changes in each hBO group were completely separated (LS mean and 95% CI), and statistically different (all *p* < 0.0001) vs. the change in the placebo group. (Table 4).

### Additional efficacy analyses

Table 5 illustrates the number of responders and non-responders for each study day and as defined by the OMERACT–OARSI responder index (Pham et al. 2004). On day 14, the number of non-responders was similar and still relatively high in all study groups (> 81%). On day 28, there was a shift in favor of responders in the hBO groups, and by study end, the % responders had shifted dramatically in favor of the hBO groups: 83.64%, 81.13%, 76.7% and 10.91%, in the hBO-HD, hBO-MD, hBO- LD and placebo groups respectively. On day 56, the distribution of responders in each hBO group was significantly different vs. the distribution in the placebo group (all *p* < 0.0001).

**Table 5:** OMERCT-OARSI responder index by visit

Visit	Responders/non-responders	HD-hBO (*N* = 55)	MD-hBO (*N* = 53)	LD-hBO (*N* = 60)	Placebo (*N* = 55)
Visit 2 (day 14)	Responders	10 (18.2%)	8 (15.1%)	8 (13.3%)	7 (12.7%)
	Non-responders	45 (81.8%)	45 (84.9%)	52 (86.7%)	48 (87.3%)
Visit 3 (day 28)	Responders	26 (47.3%)	32 (60.4%)	30 (50.0%)	19 (34.6%)
	Non-responders	29 (52.7%)	21 (39.6%)	30 (50.0%)	36 (65.5%)
Visit 4 (day 56)	Responders	46 (83.6%)	43 (81.1%)	46 (76.7%)	6 (10.9%)
	Non-responders	9 (16.4%)	10 (18.9%)	14 (23.3%)	49 (89.1%)
	*p* value	< 0.0001	< 0.0001	< 0.0001	

*p* value: day 56 difference between the distribution of OMERACT-OARSI responders (*N* [% of total]) in each hBO group vs. the distribution of responders in the placebo group (chi square test)

**Fig. 2:**
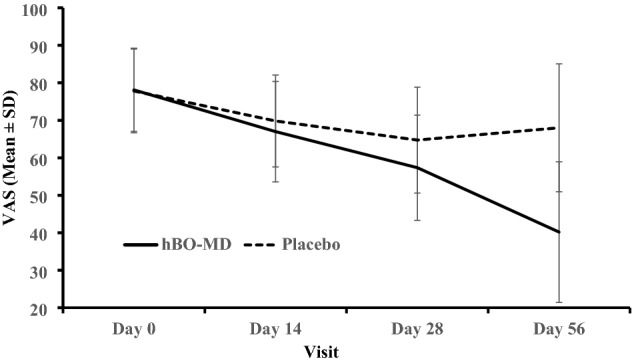
Pain VAS (mean ± SD) vs. study visit for the MD-BO and Placebo groups

To illustrate the kinetics for the onset of clinical efficacy induced by hBO, the absolute pain VAS scores (mean + SD) were plotted for the hBO-MD group, and for the placebo group at each study time point (Fig. 2). These data demonstrate a relatively slow onset of efficacy vs. pain VAS until the period between day 28 and day 56, at which point there is a more rapid increase with a study maximum decrease in the hBO-MD group recorded at study end. In contrast although there was some decrease in VAS pain in the placebo group, this had plateaued by day 28. At the primary endpoint (i.e. comparisons of the hBO and placebo groups with respect to the Baseline—day 56 changes in VAS), there was a clear differentiation in efficacy between the placebo and each hBO group (Table 3). The pain VAS kinetics were almost identical for the two other BO groups (data not shown). The kinetics for the onset of clinical efficacy kinetics was similar for the pain, function, and stiffness domains of the mWOMAC (data not shown).

There was sporadic use of rescue medication (acetaminophen) throughout the study period with no clear differences in use between groups at any timepoint.

### Safety analyses

At each study time point, all enrolled participants had mean systolic blood pressures ≤ 139 mm Hg, mean diastolic blood pressures ≤ 90 mm Hg, and pulse rates within the normal range (60–100 bpm). Blood markers of kidney (creatinine) and liver (AST and ALT) all fell within normal ranges for each enrolled patient at each study time point. There were no deaths, SAEs or other significant AEs reported during the study. AEs were reported at least once for 1 participant in the hBO-HD arm, and for 2 participants in the hBO-MD arm. There were no AEs reported in either the hBO-LD or the placebo groups. Reported AEs were all mild in nature and none had probable or definite relationships to the study IP.

## Discussion

The primary purpose of this pilot study was to understand if the clinical safety and efficacy of hBO previously demonstrated in veterinary species (Beths et al. 2020; Pearson et al. 2009; Pearson et al. 2012; Seabaugh et al. 2022; Whittem et al. 2021) would translate into decreased knee pain in a human population with X-ray diagnosed knee OA. Because of uncertainties in translating doses from animals to humans, we explored the safety and efficacy of three different doses of hBO. At the primary endpoint, all three doses of hBO resulted in changes in pain VAS that were distinct (no overlap of 95% CI) and highly significant (all *p* < 0.0001) vs. the change in the placebo group. Since statistically significant changes in measured clinical endpoints do not necessarily equate to clinical relevance, thresholds for meeting minimal clinically important improvements (MCII) have been determined in the context of hip and knee OA for both pain VAS and WOMAC function (Tubach et al. 2005). In addition, since absolute changes in pain and function are influenced by initial disease severity, MCII thresholds have been further refined based on tertiles of baseline knee pain (Tubach et al. 2005). In the current trial of hBO, the mean baseline pain VAS of approximately 78 placed this population into the high pain tertile (baseline VAS > 66.2), for which the MCII has been defined as (Mean [95% C.I.] − 36.6 (− 38.3 to − 34.7). The LS mean changes at the primary endpoint in the pain VAS scores for each hBO group, but not the placebo, all fall within the 95% CI for meeting the MCII threshold.

The exploratory endpoints are reported with *p* values uncorrected for multiple testing and are presented as nominal, requiring independent prospective replication to confirm their significance. At study end, the changes from baseline in each hBO dose for mWOMAC total and subscale scores (pain, function, stiffness), as well as the change from baseline in the SF-36 total score and each SF-36 domain score (physical function, physical health, body pain, social function, mental function, emotional health, vitality, general health) were distinct (no overlap of 95% CI) and significant (all *p* < 0.0001) vs. the change in the placebo group. Since regaining function is of high importance to OA patients, an MCII threshold has also been defined for WOMAC function (Tubach et al. 2005). For patients in the severe baseline knee pain tertile, the MCII threshold was determined to be (Mean [95% CI] − 20.4 (− 22.5 to − 18.1) on a 100-point WOMAC function scale (Tubach et al. 2005). The changes in mWOMAC function scores in the current study, normalized to a 100-point scale (Mean [95% C.I.]), were − 38.2 (− 44.3, − 32.2), − 40.9 (− 46.9, − 34.7) and − 36.3 (− 42.1, − 30.6) for the hBO-HD, hBO-MD, and hBO-LD groups respectively. These all clearly exceed the MCII threshold for improved WOMAC function. In summary, the mWOMAC function and SF-36 data provide support for hBO not only decreasing pain as per the primary pain VAS outcome, but also improving joint function and patient quality of life.

The OMERACT–OARSI responder index, developed to distinguish between an efficacious active treatment and a placebo, has been validated in 14 human clinical trials (Pham et al. 2004). In the current study the percent responders was similar in all groups on day 14; however, by day 28 the % responders in the hBO groups was beginning to differentiate from the placebo group. By day 56, the percent responders was numerically much greater in each hBO group in comparison to the placebo group (approximately 80% vs. 10.9%) and the distribution of responders in each hBO group was significantly different (nominal *p* values all < 0.0001) from the placebo group.

For each endpoint assessed longitudinally (i.e., pain VAS, mWOMAC and OMERACT-OARSI responder index), the onset of efficacy was gradual; however, between days 28 and 56, there was an accelerated accumulation of efficacy with both maximal efficacy and differentiation from placebo achieved at study end. This is illustrated in the pain VAS time course (Fig. 2) and is consistent with observations from two prior canine OA studies (Beths et al. 2020; Whittem et al. 2021) in which efficacy of hBO increased with duration of dosing.

In accordance with the observed safety profile of hBO in veterinary species and in preclinical safety studies, there were no observed safety concerns at any dose over the 56-day study. All clinical safety measures and biochemical safety measures fell within normal limits at all time points. There were no SAEs, plus the AEs reported were mild in nature and could not be ascribed to the intervention. Over the dose range explored and the 56-day study period, hBO exhibited a favorable safety profile.

Limitations to the trial include focus on a population with relatively severe knee pain at baseline, an age range limited to 40–65, and a relatively recent (< 3 years) clinical diagnosis of OA. Although the baseline pain VAS scores were relatively high (approximately 78) in the current study, they are consistent with knee OA trials conducted in India with similar patient populations (Shep et al. 2019; Srivastava et al. 2016); however, further studies would be required to understand the efficacy of hBO in populations with less severe knee pain, and in patients outside the studied age range, and who had been diagnosed with OA for periods of time > 3 year. In addition, we did not study the efficacy of hBO vs. other forms of OA (e.g., hip and hand), follow-up studies in these populations would be required. In the current trial, the placebo response (16.8% on day 28, declining to 12.6% on day 56) was about half the placebo response observed in a meta-analysis of COX-2 inhibitor trials in patients with knee pain in predominantly Western populations (Reiter-Niesert et al. 2016). Other knee pain OA trials conducted in India and based on pain VAS score outcomes, have demonstrated similar placebo responses to those in the current trial and it is possible that there are differences in pain placebo responses with respect to societal factors, and/or geography and/or ethnicity (Ramakanth et al. 2016; Sengupta et al. 2010). There were no obvious differences in the use of rescue medication in any of the hBO groups vs. the placebo group; however, there was also no clear pattern in the use of acetaminophen, and it is possible that greater reductions in use after hBO treatment might be observed in longer duration trials of hBO. Finally, although three doses of hBO were explored in the trial, no clear dose response was observed, and all three doses were similarly efficacious vs. the placebo. These data suggest that all three doses of hBO are on a plateau of maximal efficacy and that lower doses than those utilized in the current trial would need to be assessed to demonstrate lower efficacy and a clear dose response. At the study end, there were small numerical advantages in pain VAS scores in the hBO-MD vs. the hBO-LD and, given the once-a-day regimen and safety profile, this mid-dose will likely be utilized in confirmatory human studies.

Although efficacy vs. joint function and/or pain has now been demonstrated in OA trials in both canines (Beths et al. 2020; Whittem et al. 2021) and humans, neither the identity of the active species in hBO, nor the mechanism of action of hBO are clear. In both in vitro (Pearson et al. 2008) and in vivo (Pearson et al. 2009; Pearson et al. 2012; Seabaugh et al. 2022) studies, there is evidence of reductions in PGE2 after treatment with hBO. The relatively slow onset of clinical efficacy vs. joint pain is consistent with a mechanism in which membrane proinflammatory lipid precursors are cumulatively replaced with lipids that either replace proinflammatory lipid substrates, or which act via alternate mechanisms (e.g., inhibition of MAPKs) to reduce the inflammatory response (Chen et al. 2015, 2012). Interestingly it has also been demonstrated that the seed oil of another conifer species, *Sciadopitys verticillate* that contains high levels of sciadonic acid also contains a 2-monoacylglycerol, 2-sciadonylglycerol, that acts as a potent cannabinoid type 1 receptor agonist (Nakane et al. 2000). To date, it is unknown whether hBO contains 2-sciadonylglycerol, or whether this molecule is generated via metabolism after oral administration of sciadonic acid containing seed oils. Finally, there is also evidence that other mechanisms may be playing a role in the pharmacology of hBO that is of relevance to OA. In in vitro cartilage explants studies, hBO increased chondrocyte number (Pearson et al. 2008) while in a pilot equine model of surgically induced joint degeneration, hBO almost completely inhibited X-ray measured bone changes, including osteophyte growth (Seabaugh et al. 2022). It is unclear whether these activities on chondrocytes and/or bone are contributing to the observed clinical efficacy of hBO vs. joint pain and function, and whether longer term the intervention might lead to disease modifying activity. Since bone remodeling is a feature of OA (Zhu et al. 2021), and osteophytosis has been demonstrated to correlate with joint pain and/or function (Muraki et al. 2015), it is possible that hBO is also playing a role in mitigating pathological bone remodeling in joint tissues.

## Conclusions

This first human trial of hBO was designed as a pilot study; however, the trial methodology (blinded, placebo controlled, randomized) and the robustness of the intervention on the pain VAS primary outcome, plus efficacy vs. exploratory outcomes, all provide confidence that hBO has the potential to provide meaningful clinical benefit vs. knee pain. These data provide justification for further assessment of the molecular mechanism(s) of hBO, and for additional trials of hBO including independent replication, expansion into other OA populations, investigation of efficacy in joints other than the knee, and assessment of disease modifying activity.

## Data Availability

The datasets generated and analyzed during the current study are available at https://www.clinicaltrials.gov. Registration number: NCT04117490.

## References

[CR1] Alamdari DH, Aghasizadeh-Sharbaf M, Mohadjerani M, Ferns GA, Avan A (2017). Prooxidant-antioxidant balance and antioxidant properties of *Thuja orientalis* L.: a potential therapeutic approach for diabetes mellitus. Curr Mol Pharmacol.

[CR2] Bannuru RR, Osani MC, Vaysbrot EE, Arden NK, Bennell K, Bierma-Zeinstra SMA, Kraus VB, Lohmander LS, Abbott JH, Bhandari M, Blanco FJ, Espinosa R, Haugen IK, Lin J, Mandl LA, Moilanen E, Nakamura N, Snyder-Mackler L, Trojian T, Underwood M, McAlindon TE (2019). OARSI guidelines for the non-surgical management of knee, hip, and polyarticular osteoarthritis. Osteoarthr Cartil.

[CR3] Bellamy N, Buchanan WW, Goldsmith CH, Campbell J, Stitt LW (1988). Validation study of WOMAC: a health status instrument for measuring clinically important patient relevant outcomes to antirheumatic drug therapy in patients with osteoarthritis of the hip or knee. J Rheumatol.

[CR4] Beths T, Munn R, Bauquier SH, Mitchell P, Whittem T (2020). A pilot study of 4CYTE™ Epiitalis® Forte, a novel nutraceutical, in the management of naturally occurring osteoarthritis in dogs. Aust Vet J.

[CR5] Caruntu S, Ciceu A, Olah NK, Don I, Hermenean A, Cotoraci C (2020). *Thuja occidentalis* L. (*Cupressaceae*): ethnobotany phytochemistry and biological activity. Molecules.

[CR6] Chen SJ, Huang WC, Yang TT, Lu JH, Chuang LT (2012). Incorporation of sciadonic acid into cellular phospholipids reduces pro-inflammatory mediators in murine macrophages through NF-κB and MAPK signaling pathways. Food Chem Toxicol.

[CR7] Chen SJ, Chuang LT, Liao JS, Huang WC, Lin HH (2015). Phospholipid Incorporation of non-methylene-interrupted fatty acids (NMIFA) in murine microglial BV-2 cells reduces pro-inflammatory mediator production. Inflammation.

[CR8] Colletti A, Cicero AFG (2021). Nutraceutical approach to chronic osteoarthritis: from molecular research to clinical evidence. Int J Mol Sci.

[CR9] da Costa BR, Saadat P, Basciani RM, Agarwal A, Johnston BC, Jüni P (2021). Visual Analogue Scale has higher assay sensitivity than WOMAC pain in detecting between-group differences in treatment effects: a meta-epidemiological study. Osteoarthr Cartil.

[CR10] Honvo G, Bruyère O, Geerinck A, Veronese N, Reginster JY (2019). Efficacy of chondroitin sulfate in patients with knee osteoarthritis: a comprehensive meta-analysis exploring inconsistencies in randomized, Placebo-Controlled Trials. Adv Ther.

[CR11] Hughes R, Carr A (2002). Trial of glucosamine sulphate as an analgesic in osteoarthritis of the knee. Baseline.

[CR12] Jin Y, Yang HO, Son JK, Chang HW (2012). Pinusolide isolated from biota orientalis inhibits 5-lipoxygenase dependent leukotriene C4 generation by blocking c-Jun N-terminal kinase pathway in mast cells. Biol Pharm Bull.

[CR13] Kim JY, Kim HJ, Kim SM, Park KR, Jang HJ, Lee EH, Jung SH, Ahn KS (2011). Methylene chloride fraction of the leaves of *Thuja orientalis* inhibits in vitro inflammatory biomarkers by blocking NF-κB and p38 MAPK signaling and protects mice from lethal endotoxemia. J Ethnopharmacol.

[CR14] Kim TH, Li H, Wu Q, Lee HJ, Ryu JH (2013). A new labdane diterpenoid with anti-inflammatory activity from *Thuja orientalis*. J Ethnopharmacol.

[CR15] Lee YJ, Hwang SM, Yoon JJ, Lee SM, Kyung EH, Kim JS, Kang DG, Lee HS (2010). Inhibitory effect of *Thuja orientalis* on TNF-α-induced vascular inflammation. Phyther Res.

[CR16] Liu X, Machado GC, Eyles JP, Ravi V, Hunter DJ (2018). Dietary supplements for treating osteoarthritis: a systematic review and meta-Analysis. Br J Sports Med.

[CR17] Muraki S, Akune T, Nagata K, Ishimoto Y, Yoshida M, Tokimura F, Tanaka S, Kawaguchi H, Nakamura K, Oka H, Yoshimura N (2015). Does osteophytosis at the knee predict health-related quality of life decline? A 3-year follow-up of the ROAD study. Clin Rheumatol.

[CR18] Nakane S, Tanaka T, Satouchi K, Kobayashi Y, Waku K, Sugiura T (2000). Occurance of a novel cannabimimetic molecule s-sciadonoylglycerol (2-eicosa-5’,11’,14’-trienoylglycerol) in the umbrella pine Sciadopiys verticllata seeds. Biol Pharm Bull.

[CR19] Patel A, Donegan D, Albert T (2007). The 36-item short form. J Am Acad Orthop Surg.

[CR20] Pearson W, Orth MW, Karrow NA, Lindinger MI (2008). Effects of simulated digests of *Biota orientalis* and a dietary nutraceutical on interleukin-1– induced inflammatory responses in cartilage explants. Am J Vet Res.

[CR21] Pearson W, Orth MW, Lindinger MI (2009). Evaluation of inflammatory responses induced via intra-articular injection of interleukin-1 in horses receiving a dietary nutraceutical and assessment of the clinical effects of long-term nutraceutical administration. Am J Vet Res J Vet Res.

[CR22] Pearson W, Cote N, Desjardins M (2012). A dietary nutraceutical product reduces synovial fluid prostaglandin E 2 in horses with osteoarthritis: a double-blind randomized trial. AAEP Proceedings.

[CR23] Pham T, van der Heijde D, Altman RD, Anderson JJ, Bellamy N, Hochberg M, Simon L, Strand V, Woodworth T, Dougados M (2004). OMERACT-OARSI initiative: osteoarthritis research society international set of responder criteria for osteoarthritis clinical trials revisited. Osteoarthr Cartil.

[CR24] Ramakanth GSH, Uday Kumar C, Kishan PV, Usharani P (2016). A randomized, double blind placebo controlled study of efficacy and tolerability of Withaina somnifera extracts in knee joint pain. J Ayurveda Integr Med.

[CR25] Reginster JY, Veronese N (2021). Highly purified chondroitin sulfate: a literature review on clinical efficacy and pharmacoeconomic aspects in osteoarthritis treatment. Aging Clin Exp Res.

[CR26] Reiter-Niesert S, Boers M, Detert J (2016). Short-term placebo response in trials of patients with symptomatic osteoarthritis: differences between hip and knee. Osteoarthr Cartil.

[CR27] Seabaugh KA, Frisbie DD, Barrett MF, McIlwraith CW (2022). Examining the effects of an extract of Biota orientalis in the osteochondral fragment exercise model of osteoarthritis. Front Vet Sci.

[CR28] Sengupta K, Krishnaraju AV, Vishal AA, Mishra A, Trimurtulu G, Sarma KVS, Raychaudhuri SK, Raychaudhuri SP (2010). Comparative efficacy and tolerability of 5-loxinspi® and aflapinspi® against osteoarthritis of the knee: a double blind, randomized, placebo controlled clinical study. Int J Med Sci.

[CR29] Shep D, Khanwelkar C, Gade P, Karad S (2019). Safety and efficacy of curcumin versus diclofenac in knee osteoarthritis: a randomized open-label parallel-arm study. Trials.

[CR30] Shin IS, Shin NR, Jeon CM, Kwon OK, Hong JM, Kim HS, Oh SR, Ahn KS (2015). *Thuja orientalis* reduces airway infammation in ovalbumin-induced allergic asthma. Mol Med Rep.

[CR31] Srivastava S, Saksena AK, Khattri S, Kumar S, Dagur RS (2016). Curcuma longa extract reduces inflammatory and oxidative stress biomarkers in osteoarthritis of knee: a four-month, double-blind, randomized, placebo-controlled trial. Inflammopharmacology.

[CR32] Tsai PJ, Huang WC, Lin SW, Chen SN, Shen HJ, Chang H, Chuang LT (2018). Juniperonic acid Incorporation into the phospholipids of murine macrophage cells modulates pro-inflammatory mediator production. Inflammation.

[CR33] Tubach F, Ravaud P, Baron G, Falissard B, Logeart I, Bellamy N, Bombardier C, Felson D, Hochberg M, Van Der Heijde D, Dougados M (2005). Evaluation of clinically relevant changes in patient reported outcomes in knee and hip osteoarthritis: the minimal clinically important improvement. Ann Rheum Dis.

[CR34] Whittem T, Richards L, Alexander J, Beck C, Knight C, Milne M, Rockman M, Saunders R, Tyrrell D (2021). A randomised controlled masked clinical trial of two treatments for osteoarthritis in dogs. Aust Vet J.

[CR35] Wolff RL, Deluc LG, Marpeau AM (1996). Conifer seeds: oil content and fatty acid composition. J Am Oil Chem Soc.

[CR36] Yang W, Sun C, He SQ, Chen JY, Wang Y, Zhuo Q (2021). The efficacy and safety of disease-modifying osteoarthritis drugs for knee and hip osteoarthritis—a systematic review and network meta-analysis. J Gen Intern Med.

[CR37] Zhu X, Chan YT, Yung PSH, Tuan RS, Jiang Y (2021). Subchondral bone remodeling: a therapeutic target for osteoarthritis. Front Cell Dev Biol.

